# Gas Phase Reaction of Silane with Water at Different Temperatures and Supported by Plasma

**DOI:** 10.1021/acsomega.2c07209

**Published:** 2023-02-24

**Authors:** Maik Szafarska, Vinzent Olszok, Ulrich Holländer, René Gustus, Alfred P. Weber, Wolfgang Maus-Friedrichs

**Affiliations:** † Clausthal Centre of Materials Technology, Clausthal University of Technology, Leibnizstrasse 9, 38678 Clausthal-Zellerfeld, Germany; ‡ Institute of Particle Technology, Clausthal University of Technology, Leibnizstrasse 19, 38678 Clausthal-Zellerfeld, Germany; § Institut für Werkstoffkunde (Materials Science), Leibniz Universität Hannover, An der Universität 2, 30823 Garbsen, Germany

## Abstract

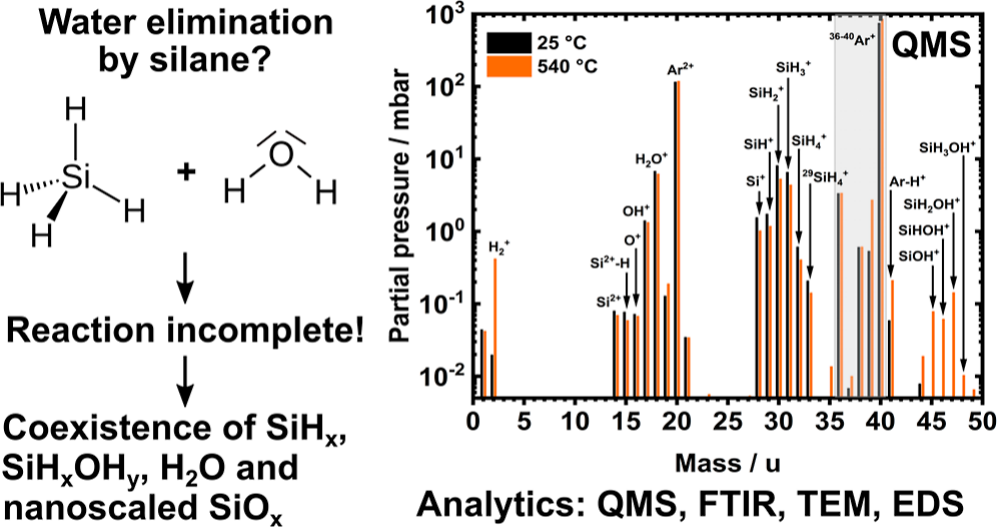

The interaction of silane and water is discussed controversially in literature: some authors suggest monosilane and water react kinetically and sufficiently fast enough to remove water, while others state the reaction occurs only at elevated temperatures. This question is of technological interest for the removal of unavoidable water residues in Ar gases. Thermodynamic calculations show that virtually complete removal of water is expected with superstoichiometric silane addition. However, mass spectrometric and infrared spectroscopic experiments give evidence that the addition of monosilane to such an Ar gas at room temperature is unable to remove residual water, which disagrees with some current hypotheses in the literature. This holds even for very high SiH_4_ concentrations up to 2 vol.-%. Silane reacts with water above temperatures of 555 °C, initiated by the thermal decomposition of silane. A cold dielectric barrier discharge-plasma used for silane and water dissociation enhances reactivity similar to elevated temperatures. Fourier-transformed infrared spectroscopy points toward silanol generation at temperatures between 400 and 550 °C, while quadrupole mass spectrometry indicates the creation of SiOH^+^, SiHOH^+^, SiH_2_OH^+^, and SiH_3_OH^+^. Cold plasmas generate smaller amounts of SiOH^+^, SiHOH^+^, and SiH_2_OH^+^ compared to elevated temperatures. Reaction products are hydrogen and nanoscaled particles of non-stoichiometric silicon oxides. The silicon-oxide particles produced differ in elemental composition and shape depending on the prevailing water content during decomposition: SiO_*x*_ generated with residual water appears with relatively smooth surfaces, while the addition of water supports the formation of significantly rougher particle surfaces. Higher initial water contents correlate with higher oxygen contents of the particles.

## 1 Introduction

The main use of monosilane is as a precursor for elemental silicon deposition within the semiconductor industry. At temperatures beyond 300 °C, silane gets chemically unstable, and decomposes into hydrogen and elemental silicon.^1−3^ Additionally, there is a second key attribute belonging to silane: it is highly reactive with oxygen, even at room temperature (RT).^4−6^ Due to this property, silane currently finds increased attention in metal processing.^7^ Metal surfaces exposed to oxygen during processing in air, even to inert gases, usually form an oxide layer.^8^ These oxide layers may have a negative effect regarding the production and processing of the metals, for example, by affecting solderability.^9^ In some cases, even a small oxide layer consisting of only a few monolayers of oxygen may inhibit the soldering of certain surfaces completely. Adding small amounts of silane into the processing atmosphere (“silane doping”) can decrease the oxygen activity inside those atmospheres way below 10^–11^ mbar—levels otherwise only found in an extremely high vacuum (XHV) in extraterrestrial space.^10^ The two reaction products, hydrogen and silicon dioxide, either have a reductive character or are highly unreactive, thus preventing the formation of an oxide layer. However, depending on the metal, the residual water within the processing atmosphere will probably also have an oxidizing effect on the surface.^11,12^ In literature, it is frequently suggested that silane reacts with water at RT efficiently and kinetically fast enough to remove residual water in processing atmospheres.^13^ Unfortunately, there was no experimental evidence provided along with the statements, making it impossible to reproduce any experiments. The following reaction equation is often quoted when stating that the stoichiometric doping of the atmosphere with silane is suitable to remove the residual water content of the atmosphere:
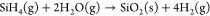
1

A recently published work by the authors of this article discussed the transport of pure titanium samples in silane-doped inert gas atmospheres over an extended time in order to prevent oxidation of the titanium surface. Contrary to expectations following eq 1, after several hours passing, the titanium samples showed small signs of oxidation even in silane-doped argon with XHV-equivalent oxygen activities, confirmed by an oxygen sensor.^14^

Other studies have found silane hydrolysis to be kinetically slow at RT, with silanols as the reaction product rather than SiO_2_.^15,16^ Mitsui et al. determined the amount of trace water in pure monosilane to be about 20 ppb at RT.^17^ While this may appear to be a low value, it corresponds to a water partial pressure of 2 × 10^–5^ mbar at an absolute pressure of 1000 mbar. This remaining amount of water suffices to oxidize certain metal surfaces within several seconds.

Despite intense research, the authors of this article failed to find literature of experimental nature confirming the reaction in eq 1 for RT. Only a theoretical work by Hu et al. suggests SiO as a product of silane hydrolysis.^18^ However, the authors state the reaction to occur at elevated temperatures, not RT. The exact temperature needed for the reaction of SiH_4_ and H_2_O with SiO as product species remains unknown. There exists no experimental evidence of SiO_2_ as a direct product of silane or silicon monoxide hydrolysis in literature. Given the scarcity of experimental research regarding the silane–water gas phase behavior, this article aims to clear up some misconceptions and answer the following questions: Is silane suitable to remove residual water in silane-doped argon atmospheres at RT? How does temperature influence the reactivity and reaction products between silane and water? What are the reaction products of the monosilane and water reaction if they do react?

## 2 Experimental Section

### 2.1 Setup

**Figure 1:**
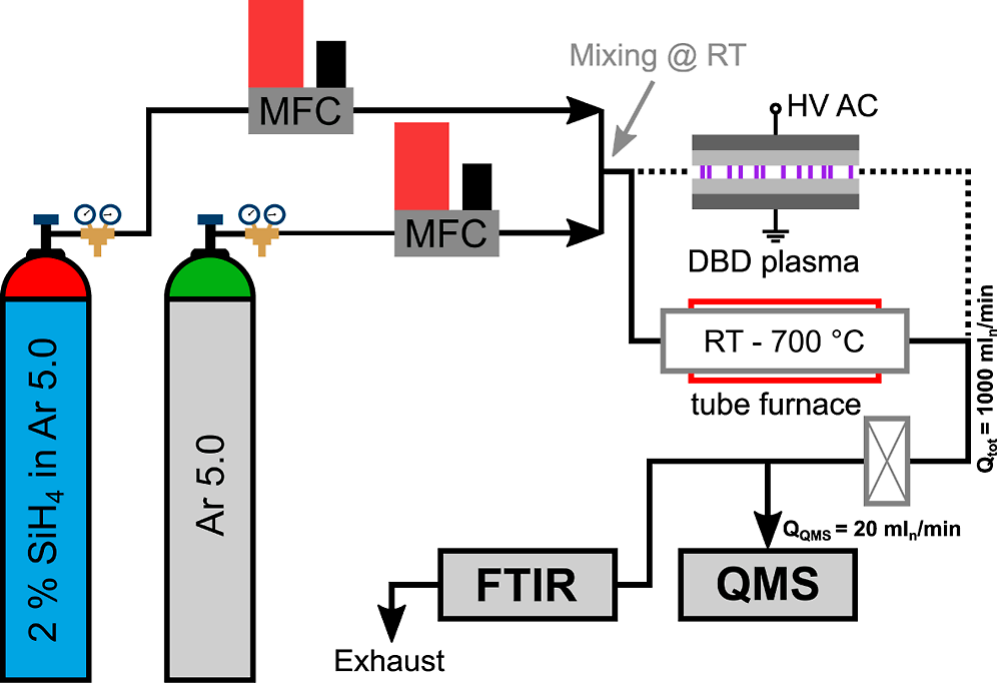
Experimental setup: the gas mixture passes either a tube furnace (solid line, *L* = 65 cm) or a DBD plasma (dashed line, *L* = 10 cm) followed by an absolute filter for particle removal before the gas enters the analytical stage (QMS and FTIR). An optional water vapor source (gas washing bottle) can be integrated into the Ar 5.0 line before mixing occurs at RT.

Figure 1 shows the deployed experimental setup and the interconnection of the two analytical tools, quadrupole mass spectrometry (QMS) and Fourier-transformed infrared spectroscopy (FTIR), respectively. Two thermal mass flow controllers (MFC, el-flow, Bronkhorst Deutschland Nord GmbH) controlled argon and argon–silane gas flow during measurement. The argon used had a purity of 99.999% (5.0, Linde AG), and monosilane had a purity of 99.99%. The monosilane was a premixed gas with 2 vol.-% silane in argon 5.0 (Linde AG). The total flow rate passing through the setup was constant at 1000 mL_n_/min. Downstream the point of gas mixing, a tube furnace was set up, allowing an interaction between silane and water at elevated temperatures with a residence time of 12 s at RT and 4 s at 700 °C. The water either originates from the used pure argon (trace amounts), desorbed water from the gas tube surfaces, or from an argon–water saturator inserted into the argon line behind the MFC. A gas washing bottle filled with degassed and deionized water was used to preload the argon with larger amounts of gaseous water, if desired. Argon with about 80% RH was used in all of the experiments with added water. A dielectric barrier discharge (DBD) plasma was applied to investigate the plasma-enhanced reactivity between silane and water. The sinusoidal plasma voltage was 12 kV_PP_, while the frequency was set at 108.6 kHz. As previously stated, a reaction between silane and oxygen, or silane and water, produces solid SiO_*x*_, which is intended to be filtered by an absolute filter downstream the tube furnace before the gaseous components enter the analytical stage. SiO_*x*_ particle collection was done by sampling from the filter. In order to keep the delay between both spectrometers at a minimum of about 2 s, the length of the gas tubing was kept as short as possible downstream the furnace.

### 2.2 Spectroscopy Methods for Gas Phase and Particle Analysis

Gaseous components were tracked using a quadrupole mass spectrometer (QMS, Cirrus 3, MKS Instruments). Ions are created via electron impact ionization by an open ion source within the QMS. The ionization energy was at a constant 70 eV during all experiments. The chamber pressure remained constant at 5 × 10^–6^ mbar inside the QMS. For atmospheric gas composition analysis, the pressure adjustment happens by passing the gases through a 2 m-long capillary into a chamber evacuated by a turbomolecular pump with a total pressure gradient of 5 × 10^8^ mbar measured from the entrance. According to the manufacturer, the capillary is non-selective for molecule size and heated at a constant temperature of 180 °C. Because monosilane and water exhibit a distinct dipole moment, FTIR (Tensor 27, Bruker) measurements were applicable in parallel to QMS. Transmission electron microscopy (TEM, JEM-2100, Jeol) and energy-dispersive X-ray spectroscopy (EDS, X-Max 80T, Oxford) were used as offline analytics for SiO_*x*_ particles.

## 3 Results and Discussion

### 3.1 Reactivity of Monosilane with Water at Room Temperature

**Figure 2:**
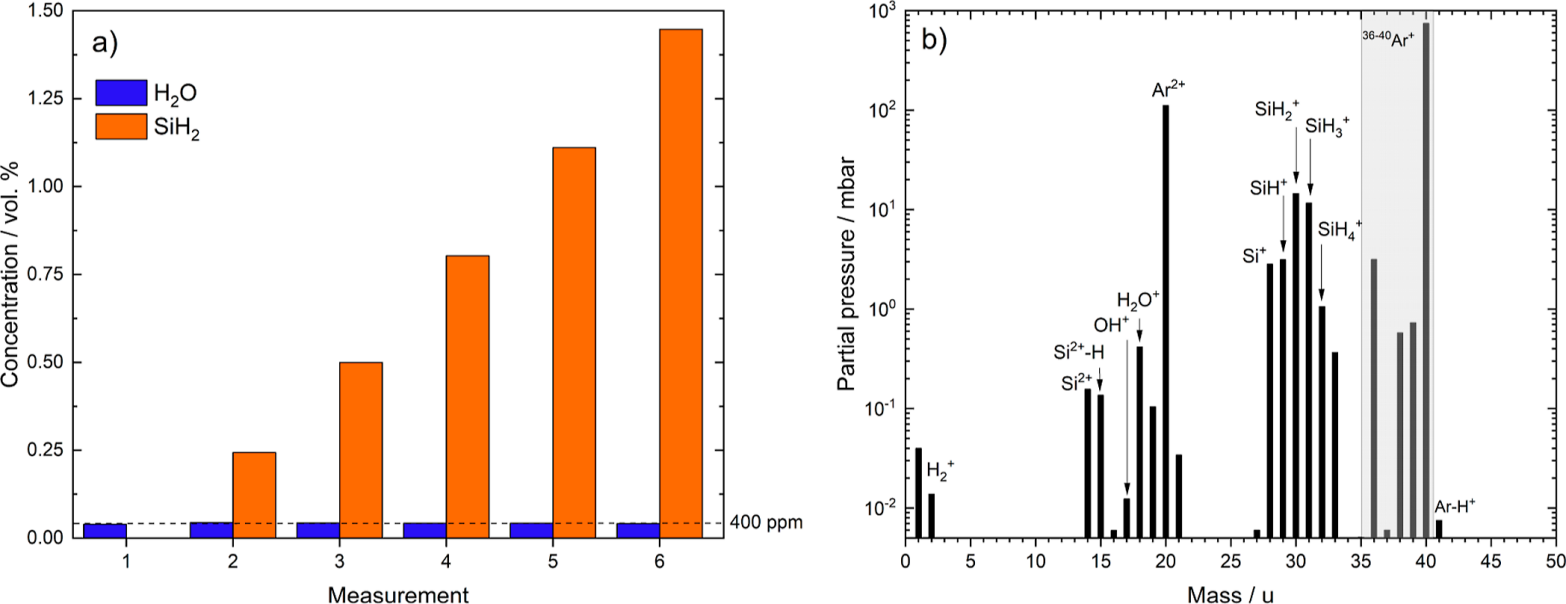
(a) Change of water content (blue) inside a silane-doped argon gas stream with varying amounts of silane added (orange). The SiH_2_-fragment signal in (a) represents the amount of silane added as silane mainly fragments into SiH_2_ during electron impact ionization. The total flow rate was at a constant 1000 mL_n_/min. (b) Mass spectrum (log-plot) of an Ar–SiH_4_ (2 vol.-%) mixture with a flow rate of 1000 mL_n_/min. Data in both graphs generated via quadrupole mass spectrometry with an ionization energy of 70 eV.

In all experiments shown, the total flow rate remained at a constant 1000 mL_n_/min while adding varying amounts of silane to an argon gas flow. Most of the remaining water likely originates from the desorption of small amounts of H_2_O off surfaces like hoses and valves. Figure 2a shows the water concentration inside the gas stream with respect to the SiH_2_ concentration. The figure displays the SiH_2_ content instead of SiH_4_ because SiH_4_ overlaps with O_2_ at mass 32 u and monosilane mainly fragments into SiH_2_ at mass 30 u without interferences originating from other masses.^19,20^ The orange bars represent the SiH_2_ concentration in ppm, while the blue bars show the residual water content 10 min after reaching the target silane concentration. In the range of 0 to 1.4 vol.-% SiH_2_, the remaining water content remains constant. Figure 2b shows the coexistence between monosilane and water in the gas phase, as the masses of both silane and water remain in the mass spectrum.

The results suggest that doping argon with silane does not decrease the water content significantly at RT. These findings are in disagreement with some claims made in other scientific articles.^21^ Even when the silane concentration is multiple times higher than the water content, only small changes in the mass spectra occur. The water content remains constant regardless of the added amount of silane, as shown in Figure 2a. This experiment supports the theory of other articles stating that the silane–water reaction is kinetically slow at RT.^22,23^ Kondo et al. reported that water remains adsorbed on the surfaces of silane combustion products. Our observation supports those made by Kondo et al. Via X-ray photoelectron spectroscopy, we observed that even small amounts of residual water affect the surface oxidation of pure titanium samples resulting in an increased surface oxide layer after several hours of exposure time, even in oxygen-free atmospheres. The remaining water in silane-doped atmospheres could explain the oxidation of the titanium surfaces discussed.

**Figure 3:**
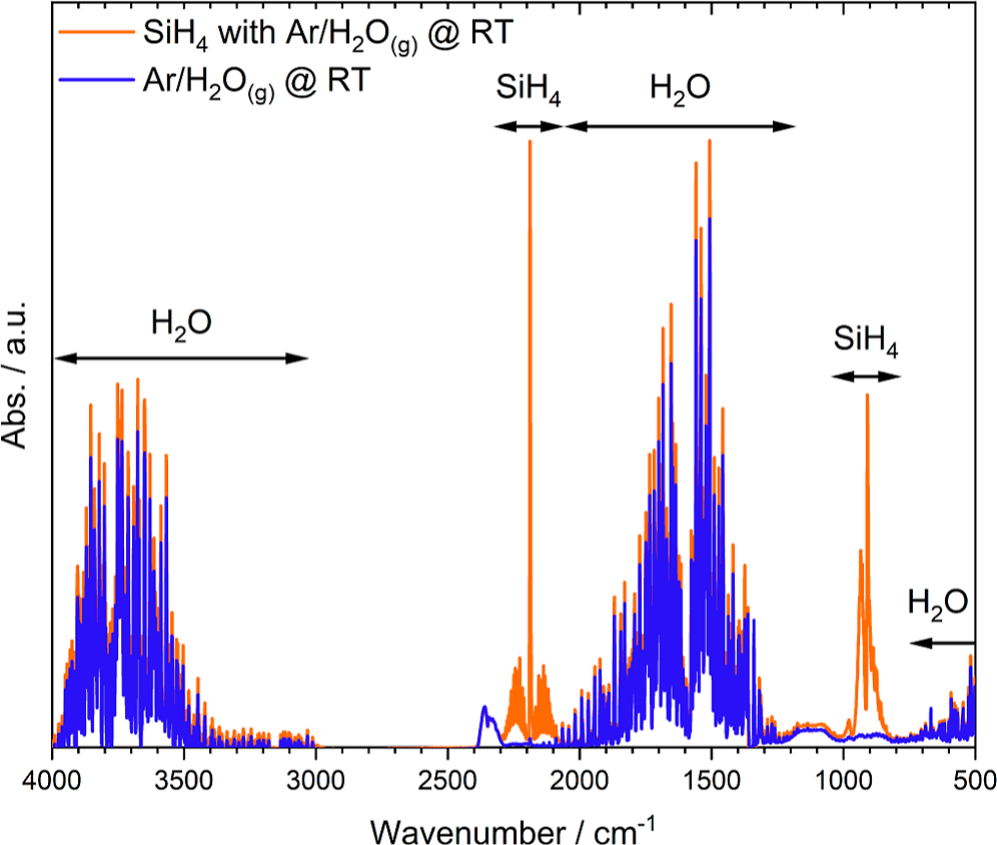
Exemplary FTIR spectra of an argon–water–silane mixture at RT showing the coexistence of water and silane. The total gas flow was constant at 1000 mL_n_/min during measurement of both spectra.

FTIR measurements further suggest a slow reaction between silane and additional water at RT, as shown in Figure 3. The blue curve refers to the FTIR response for water-loaded argon. As argon is IR inactive, the spectra only show reflexes belonging to water between 4000 and 3000, 2000 and 1300, and <700 cm^–1^. The orange spectrum shows the resulting FTIR reflexes of an argon–water–silane mixture instead of water-loaded argon. The corresponding reflexes after 10 min of constant flow through the FTIR show the characteristic silane peaks at around 2190, and 900 cm^–1^ of which the former also shows a relatively symmetrical shape.^24,25^ The arrows mark gas specific reflexes present in both measurements.

The coexisting water and silane reflexes in the orange FTIR spectrum indicate that silane and water are highly unreactive at RT. A reaction between silane and water can be precluded based on the shown FTIR spectra, since the water reflexes do not decrease significantly when silane is admixed at RT. Furthermore, the water concentration in argon–water–silane is slightly higher in contrast to pure argon–water. The slight water surplus could be a byproduct of the reaction between silane and oxygen, as shown in eq 2. In contrast to the reaction with water, the literature provides a lot more information regarding silane and oxygen reactivity.^26−30^ This is because silane is highly pyrophoric and therefore explosively reacts with oxygen according to eqs 2 and 3, which makes it of high interest for safety research and physical vapor deposition of silicon-oxides.^31−33^
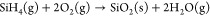
2
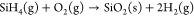
3

### 3.2 Reactivity of Monosilane with Water at Elevated Temperatures

The experiments shown in this section discuss silane and water reactivity at higher gas temperatures up to 700 °C. According to literature, monosilane thermally decomposes at temperatures between 300 and 550 °C depending on the presence of a catalytic surface, the remaining hydrogen content inside the atmosphere, and the residence time inside the reactor chamber.^34,35^ Perhaps it is possible to force a hydrolysis between water and silicon radicals formed during the thermal decomposition of silane, thus reducing the water content in the gas phase. To test this hypothesis and the temperature behavior of the silane water reactivity, silane was passed through a quartz glass tube inside a tube furnace and afterwards into the QMS and FTIR, respectively.

**Figure 4:**
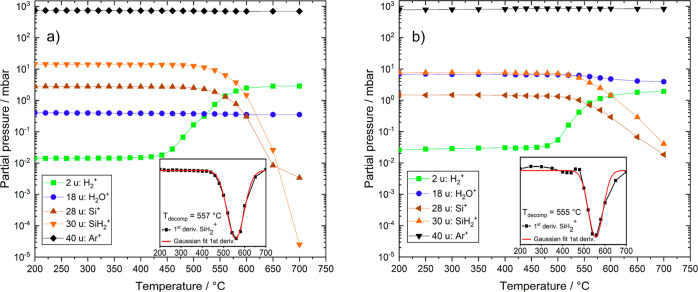
Effect of temperature on gas concentration (log-plots) of (a) an argon–silane mixture with about 400 ppm residual water content and (b) an argon–silane–water mixture of about 7000 ppm water.

The QMS results in Figure 4a show that at about 555 °C, the concentration of SiH_2_ drops below 10^–4^ mbar (<100 ppb), the detection limit of the quadrupole mass analyzer. At the same time, the hydrogen concentration increases by more than 2 orders of magnitude while the water concentration decreases by roughly 5%. The simultaneous increase in the hydrogen signal as well as the decrease in SiH_2_^+^ concentration indicate the thermal decomposition of silane. The decreasing silicon signal may be surprising at first, as silicon is supposed to be a direct product of silane decomposition. However, the silicon created this way tends to nucleate at a very fast rate into nanoscaled particles (see Section 3.5). This behavior makes it impossible to detect them with the experimental setup used in this work, by means of QMS and FTIR. Because of the slight decrease in water content at the same time, some reactive species, such as silicon radicals, probably react with water to create solid-state SiO_*x*_. Section 3.5 discusses the EDS and TEM results of the generated particles.

Figure 4b shows the resulting changes in gas composition after increasing the water content to 7000 ppm, about the same concentration as silane. Surprisingly, the water concentration falls by about 38% after surpassing the critical temperature of 555 °C for thermal silane decomposition. It is evident that changing the ratio between the amount of silane and water offered during heating can have an impact on water elimination. The probability of a reactive silane combustion fragment encountering a water molecule is most likely higher in the experiment, as shown in Figure 4b compared to Figure 4a. Nevertheless, the water concentration in the gas stream remains higher compared to the experiment with 400 ppm residual water. Compared to the findings at RT, merely increasing the temperature seems to be insufficient regarding complete water elimination via silane.

**Figure 5:**
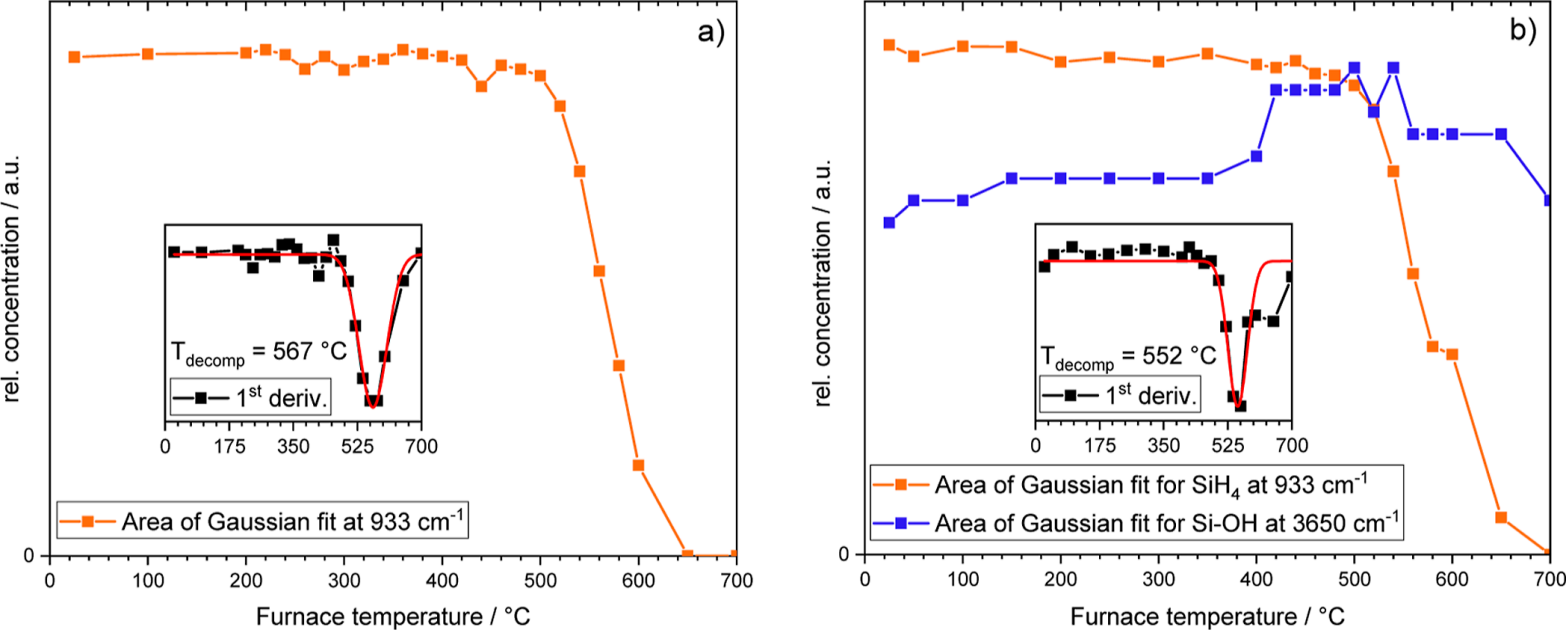
Peak area (lin-plot) vs furnace temperature obtained by FTIR analysis: (a) argon–silane, (b) argon–water–silane. The insets show the first derivation of the area vs temperature plot and give access to the decomposition temperature of silane.

The FTIR measurements shown in Figure 5a verify the thermal decomposition of silane. As silanol complexes might form by electron impact ionization during QMS measurements because of fragment recombination, FTIR is advantageous here as well. This is because QMS measurements may reflect the occurrence of silanols, although no silanols occur within the process. The FTIR analysis is a non-invasive technique that is able to detect even small amounts of silanols or other water–silane complexes that may form in the process itself. However, as exemplarily shown in Figure 3, no silanol complexes at distinct other wavenumbers compared to the reflexes caused by SiH_4_ and H_2_O, respectively, appear at RT and at elevated temperatures in the FTIR spectra for T > RT (not shown here). Nevertheless, the evolution of the peak area of certain silane peaks, and hence the concentration, with increasing temperature gives comparable data that reflect the decomposition of silane in an argon–silane mixture (see Figure 5a). A first noticeable decrease in silane concentration becomes visible at around 500 °C, based on the first derivation of the red curve as an inset. Pure silane decomposes accordingly at a temperature of 567 °C.

**Figure 6:**
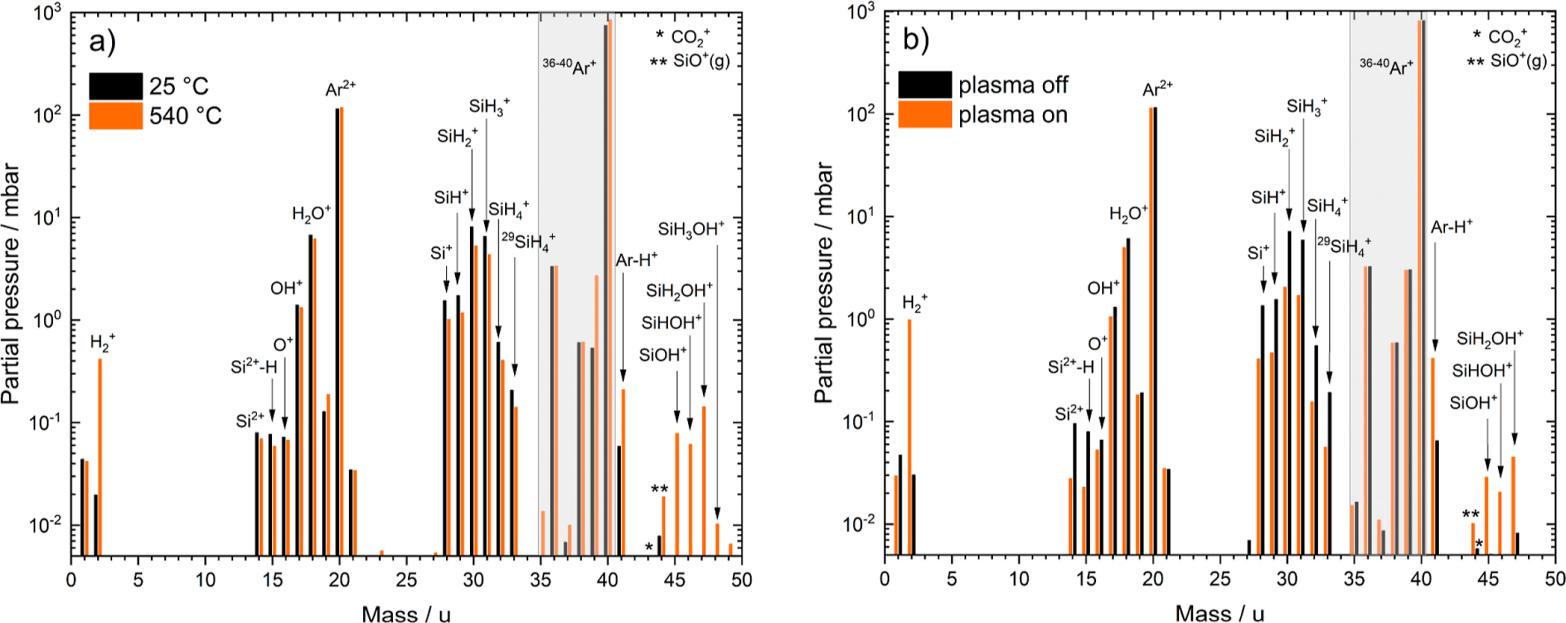
(a) Effect of elevated temperature vs RT on gas concentration (log-plot). (b) Effect of a non-thermal DBD plasma on the gas concentration (log-plot) of an argon–silane mixture with ca. 7000 ppm water. Both spectra show the generation of silanols either at higher temperatures or in a DBD plasma. Plasma parameters: voltage: 12 kV_pp_, frequency: 108.6 kHz. Both spectra were measured via quadrupole mass spectrometry with an ionization energy of 70 eV and at a constant gas flow of 400 mL_n_/min Ar–H_2_O + 600 mL_n_/min Ar–SiH_4_. The water concentration remained constant at 7000 ppm.

As stated in literature, absorption signals at 3650 cm^–1^ can be attributed to Si–OH bonding.^36^ At this point, it is important to mention that both dipoles, H_2_O and Si–OH contribute to an absorption at 3650 cm^–1^ as can be confirmed by FTIR measurements without silane. Agreeing with the decreasing water signal in Figure 5b (QMS), the FTIR showed an increased signal for Si–OH over temperature (see Figure 5b, blue curve) when silane starts to slowly decompose at around 400 °C. Hence, a water–silane interaction becomes noticeable by Si–OH detection, but only at elevated temperatures. However, complete water removal is not feasible. As explained above, the first derivation of the silane area signal over temperature gives access to the decomposition temperature that changed to lower temperatures (552 °C) when water is apparent during heating. The interaction of water and silane by means of Si–OH transition states might support the thermal decomposition of silane, as depicted by a lower decomposition temperature. Figure 6a further supports the thesis of silanol generation as there is a noticeable increase of the masses 45–48 u at 540 °C (orange) compared to RT (black). These masses most likely belong to the silanols SiOH^+^, SiHOH^+^, SiH_2_OH^+^, and SiH_3_OH^+^ in increasing mass order.

### 3.3 Thermodynamic Analysis of the Gas Phase Reactivity between Monosilane and Water

Thermodynamic calculations were performed using the commercial software FACTSAGE (GTT Technologies, Herzogenrath, Germany) to identify possible reaction products between SiH_4_ and H_2_O under the set process conditions. The calculations are based on a database in which the free enthalpies of formation of numerous hypothetical reaction products that can be formed from Si, O, and/or H are filed as a function of temperature. Input parameters are the set gas system (initial composition of mixed Ar–SiH_4_–H_2_O) and the given temperature and pressure (here standard atmospheric pressure of 101,325 Pa). All reactants and reaction products were considered in virtually all states of aggregation without suppressing certain states. Considering the conservation of the initial quantities of element atoms, in a numerical calculation procedure, the quantity of each reactant and product in the gas mixture is then adjusted so that the sum of their free enthalpies of formation is minimal. This represents the condition for unrestricted thermodynamic equilibrium in a multicomponent system.

**Figure 7:**
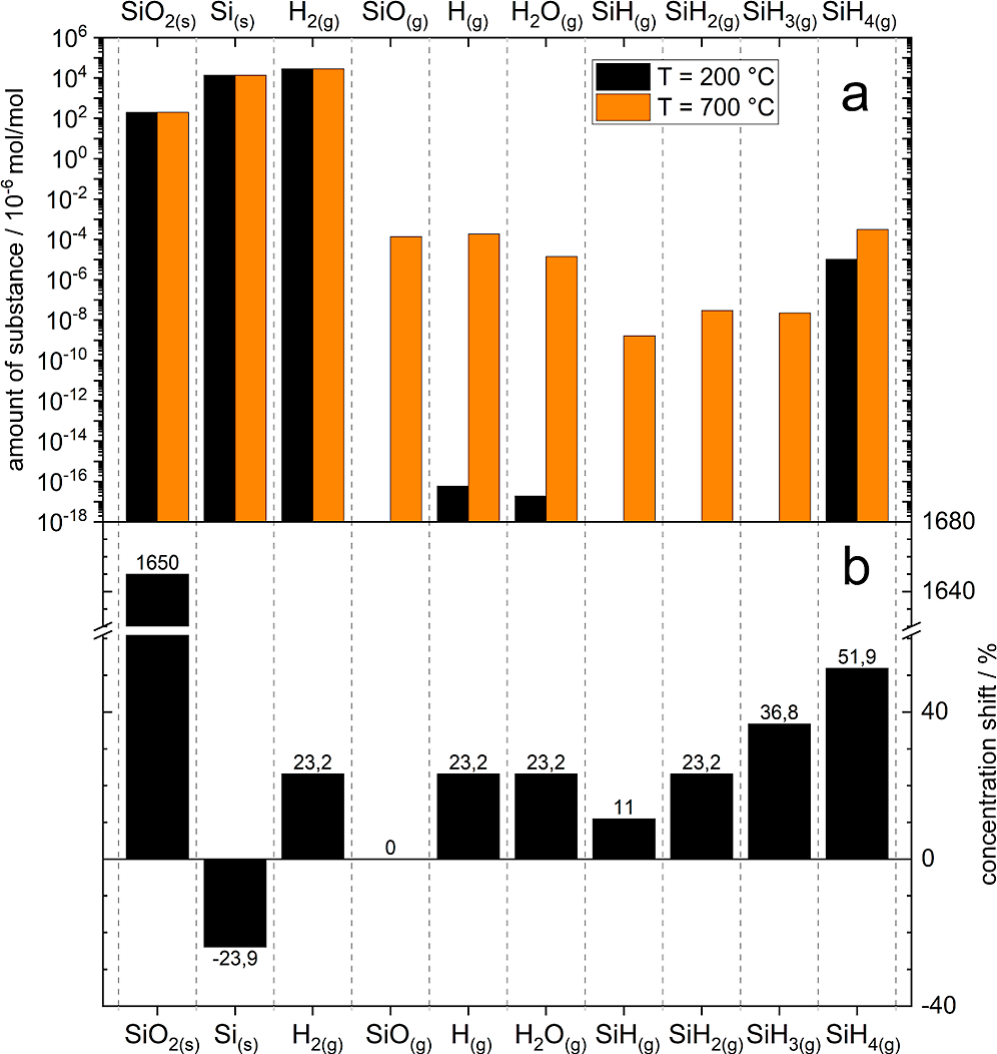
(a) Modeled equilibrium concentrations of relevant reactants and products for an initial composition of 1.4 vol.-% SiH_4_(g) and 400 ppm_v_ H_2_O(g) in argon at 200 and 700 °C and (b) relative shift of concentrations with increase of initial H_2_O from 400 to 7000 ppm at 700 °C.

In Figure 7a, the calculated equilibrium composition of a set argon mixture containing 1.4 vol.-% SiH_4_ and 400 ppm H_2_O is given. Only reaction products whose calculated concentrations reach at least 10^–24^ mol/mol (corresponding to 10^–18^ ppm_v_ in the case of gaseous products) and thus can be present at all in a typical reaction volume (1 m^3^) are considered in the bar chart. Note that the majority of the products occur at a concentration below 10^–9^ mol/mol (1 ppb) and are thus practically undetectable. Only the main products SiO_2_(s) and H_2_(g), which are to be expected according to eq 1, as well as Si(s) resulting from a postulated thermal decomposition of the excess SiH_4_, which is known as metastable for temperatures <300 °C, occur with significant concentration.

Figure 7b shows the influence on the reaction equilibrium when increasing the initial water content from 400 to 7000 ppm in the process gas. As expected, the concentration of solid silicon is reduced due to the remaining lower excess silane (measured by the water content) and simultaneously the hydrogen content from the additionally converted water is increased. The latter in turn has an influence on remaining H_2_O and volatile SiH_*x*_ residues, which all increase in their still extremely low equilibrium concentration due to the higher hydrogen supply.

In summary, the analysis of the considered gas mixtures shows that from a purely thermodynamic point of view, a virtually complete removal of the water with superstoichiometric SiH_4_ addition is to be expected even at moderate temperature. Besides gaseous hydrogen, the main reaction products are solid SiO_2_ and Si. The latter do not necessarily have to arise as separate phases but can also occur as superficially oxidized silicon particles in which both elemental silicon and silicon oxide are present (see Section 3.5).

### 3.4 Interaction of Monosilane with Water in Plasma

This section discusses whether cold DBD plasmas can enhance the reactivity between silane and water. Figure 1 shows the experimental setup with the DBD. The gas flow rate passed through the reactor was 400 mL_n_/min argon–water and argon–silane with a constant flow of 600 mL_n_/min. Figure 6b shows the resulting changes in gas partial pressure of specific gaseous components before and during active plasma, measured via mass spectrometry.

After igniting the plasma, the SiH_2_ content drops by about half an order of magnitude to about 2 mbar or 2000 ppm, respectively. Si and H_2_O concentrations drop slightly, while the hydrogen content increases by almost 2 orders of magnitude. The plasma most likely breaks down silane into atomic and molecular hydrogen, as well as silicon, silicon hydrides, and water into atomic and molecular fragments. Silicon partial pressure drops as well, most likely caused by the deposition of silicon onto the plasma electrode surface. The components SiOH^+^, SiHOH^+^, and SiH_2_OH^+^ probably cause the gain in partial pressure of the masses 45–47 u. As the hydrogen content rises, some of the H_2_^+^ produced will almost certainly contribute to the formation of the silanol species. However, compared to elevated temperatures as shown in Figure 6a, there is no contribution to the SiH_3_OH^+^ species at mass 48 u. In agreement with the former experiments using additional water during decomposition within a furnace, the plasma in combination with silane also shows the ability to remove water. However, no increased water reduction was observable with the plasma parameters used in this study. In conclusion, Si–OH species became detectable by QMS, as well as by FTIR at 3650 cm^–1^ (shown in Figure 5b). The presented DBD plasma experiments act as preliminary experiments that need expansion by a detailed study in the future.

### 3.5 Evaluation of SiO_*x*_ Formation under Different Conditions

**Figure 8:**
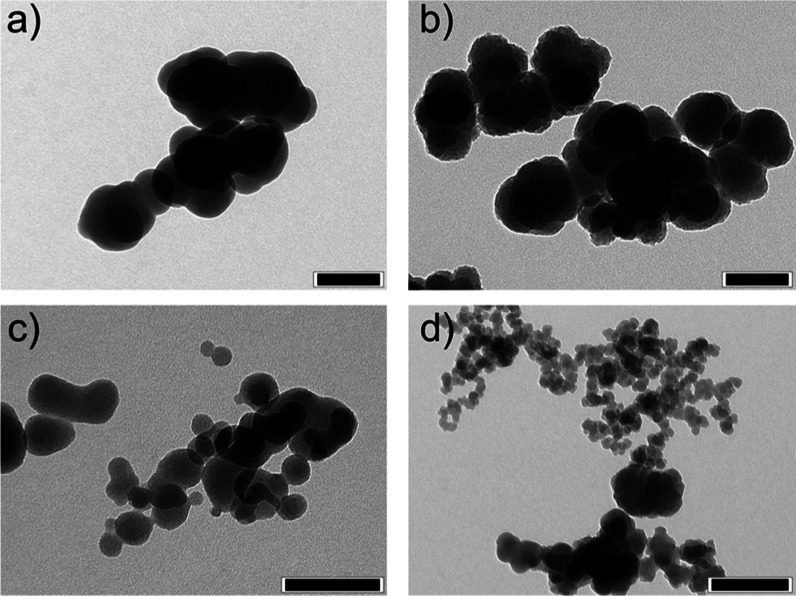
TEM micrographs of synthesized SiO_*x*_ particles: (a) pure SiH_4_ in furnace, (b) SiH_4_ and H_2_O in furnace, (c) SiH_4_ and H_2_O in DBD, and (d) SiH_4_ at exhaust. Scale bar: (a–c) 100 nm and (d) 200 nm.

Chemical eqs 1–3 refer to the production of solid silica (SiO_2_) that appears by means of nanoscaled particles. In this regard, an absolute filter in the gas/aerosol line protects the analytical stage in the experiments from damage and prevents blocking by solid silica. Transfer of the silicon oxide powder from the filter onto TEM carbon grids took place after silane decomposition at 700 °C within the furnace and at RT within the DBD plasma, respectively. At the exhaust of the experimental setup, silane reacted with ambient air, forming silica. Imaging and analysis of all four types of silica happened via TEM and EDS. Table 1 refers to the experimental conditions that led to silica formation. Interestingly, the color of collected powder varied from brown over yellow to the expected snow-white known from SiO_2_. The correlation of the elemental composition of the produced SiO_*x*_ nanoparticles with their optical appearance explains the different colors. While the brown powder is made of non-stoichiometric silica (silicon overweighs), the white powder at the exhaust of the system exhibits the expected atomic ratio of silicon to oxygen of 1:2. The yellow color of SiO_*x*_ that forms within the DBD surprises as the amount of silicon is higher than in SiO_*x*_ formed within the furnace that exhibits a brown color. Yellow silica may form due to the short residence time within the DBD and the plasma streamers, respectively. DBD plasmas, known as “cold plasmas”, however, can reach temperatures up to hundreds of Kelvin locally in a streamer.^37^ Activated gas species emerging from the plasma may cause a cold silane dissociation, hence a decomposition, induced by ionized gas molecules. Figure 8 shows the morphology of the four different types of amorphous SiO_*x*_. The TEM micrograph (c) shows the SiO_*x*_ from the DBD. In comparison, the relatively small particles support the idea of extremely fast particle formation within the DBD. The consequence might be a yellow SiO_*x*_ that represents a transition from a gaseous precursor (monosilane) to a non-stoichiometric silicon oxide.

**Table 1:** TEM and EDS Analyses of Synthesized SiO_*x*_ Particles: Differences in Experimental Conditions Result in Various Nanoparticle Colors, Morphologies, and Compositions

experimental conditions	powder color	EDS (at.-% Si, at.-% O)	comment
(a) pure SiH_4_ in furnace	brown	97, 3%	smooth particle surface
(b) SiH_4_ and H_2_O in furnace	brown	75, 25%	rough particle surface
(c) SiH_4_ and H_2_O in DBD	yellow	81, 19%	polydisperse, irregular morphology but smooth particle surface
(d) SiH_4_ at exhaust (ambient air)	white	33, 67%	fractal-like structures, highly polydisperse, smooth and rough surfaces noticeable

Furthermore, TEM and EDS analysis from Table 1 and Figure 8 support the idea of a silane–water interaction since different SiO_*x*_ synthesis conditions led to different elemental compositions and various particle morphologies. The high amount of silicon and low level of oxygen in sample (a) support the hypothesis of thermally induced silane decomposition without any interaction of oxygen-containing substances. An intended increase in water content during reaction/decomposition (b) led to an increased oxygen level in the sampled SiO_*x*_; however, there are no indications of stoichiometric SiO_2_ formation. It is unclear whether pure silicon particles form within the furnace that react with water afterward, or if silane reacts with water forming unevenly structured SiO_*x*_ nanoparticles. FTIR measurements support the idea that silane–water interaction results in a decreased decomposition temperature (15 K lower) for silane. Then, the particles shown in micrograph (b) refer to SiO_*x*_, which is formed by a silane–water reaction. Beyond that, literature refers to the possible thermal dissociation of solid SiO_2_ at temperatures above 1400 °C, the subsequent formation of silanols if water is apparent, and the re-nucleation of nanoscaled SiO_2_, supported by mass spectrometric measurements.^38,39^ These findings back up our hypothesis of a water–silane interaction that resulted in SiO_*x*_ nucleation with silanols as an intermediate product of SiH_4_ decomposition since silanols seem to appear as an intermediate product during SiO_2_ formation, independent of whether SiO_2_ or SiH_4_ is thermally decomposed. However, our results underline again that the expected scavenger capability of silane for water does not apply in practice. For oxygen, nonetheless, silane scores as a highly effective scavenger since this reaction yields stoichiometric and white SiO_2_ with a large variety in morphology (d).

## Conclusions

Results from mass spectrometry and Fourier-transform infrared spectroscopy show that silane does not eliminate water completely at RT as both components coexist in the gaseous phase. These experimental results disagree with some current theses in literature suggesting that silane can eliminate water effectively and kinetically fast to establish water-free inert gas atmospheres with SiO_2_ as a reaction byproduct. They do, however, support the findings of other sources that report on the formation of small amounts of silanols and non-stoichiometric SiO_*x*_, which our data suggest as well. It is possible that the silanols and SiO_*x*_ found in the experiments are intermediate products of a reaction pathway to SiO_2_ which did not take place until completion in the experiments. In contrast, thermodynamic analysis suggests that a virtually complete removal of water is to be expected with superstoichiometric silane addition with SiO_2_ and H_2_ as main products. Silane in a mixture of 98 vol.-% argon and 2 vol.-% silane thermally decomposes quickly between 552 and 567 °C when passing both gases through a tube furnace and heating them up to 700 °C. There are no signs of increased residual water elimination after the thermal decomposition of silane. However, after increasing the water content in the gas mixture to a ratio of 1:1 to silane, a slight drop in water concentration by about 38% beyond temperatures of 555 °C takes place. Indeed, using FTIR, there was an observable water–silane interaction showing the formation of small amounts of Si–OH species contributing to the decomposition of small amounts of silane at temperatures higher than 400 °C. First experiments on the elimination of water by silane in a DBD plasma with the parameters used in this study showed comparable results to elevated temperatures in a tube furnace. Electron microscopic analysis of the byproduct from the silane decomposition pointed, on the one hand, at the formation of solid aerosol nanoparticles. On the other hand, the evaluation of SiO_*x*_ nanoparticles with respect to their elemental composition offers a chance to investigate the reaction history of a silane molecule, since different reaction conditions led to differently shaped and compounded silicon oxides.

## Supporting Information Available

Respective EDS spectra of the synthesized SiO_*x*_ particles (PDF)

## Notes

The data that support the findings of this study are available from the corresponding author upon reasonable request.

## References

[ref1] Timms P. L. The chemistry of volatile waste from silicon wafer processing. J. Chem. Soc., Dalton Trans. 1999, 815–822. 10.1039/A806743K.

[ref2] Babushok V. I.; Tsang W.; Burgess D. R.; Zachariah M. R. Numerical study of low- and high-temperature silane combustion. Symp. (Int.) Combust. 1998, 27, 2431–2439. 10.1016/S0082-0784(98)80095-7.

[ref3] Hogness T. R.; Wilson T. L.; Johnson W. C. The Thermal Decomposition of Silane. J. Am. Chem. Soc. 1936, 58, 108–112. 10.1021/ja01292a036.

[ref4] Hartman J. R.; Famil-Ghiriha J.; Ring M. A.; O’Neal H. E. Stoichiometry and possible mechanism of SiH4-O2 explosions. Combust. Flame 1987, 68, 43–56. 10.1016/0010-2180(87)90064-2.

[ref5] Quandt R. W.; Hershberger J. F. Kinetics of the SiH3 + O2 and SiH3 + NO2 reactions. Chem. Phys. Lett. 1993, 206, 355–360. 10.1016/0009-2614(93)85564-5.

[ref6] Yang T.; Thomas A. M.; Dangi B. B.; Kaiser R. I.; Mebel A. M.; Millar T. J. Directed gas phase formation of silicon dioxide and implications for the formation of interstellar silicates. Nat. Commun. 2018, 9, 774 10.1038/s41467-018-03172-5. 29472549 PMC5823853

[ref7] Maier H. J.; Herbst S.; Denkena B.; Dittrich M.-A.; Schaper F.; Worpenberg S.; Gustus R.; Maus-Friedrichs W. Towards Dry Machining of Titanium-Based Alloys: A New Approach Using an Oxygen-Free Environment. Metals 2020, 10, 1161 10.3390/met10091161.

[ref8] Hallberg R. T.; Ludvigsson L.; Preger C.; Meuller B. O.; Dick K. A.; Messing M. E. Hydrogen-assisted spark discharge generated metal nanoparticles to prevent oxide formation. Aerosol Sci. Technol. 2018, 52, 347–358. 10.1080/02786826.2017.1411580.

[ref9] Zähr J.; Ullrich H.-J.; Oswald S.; Türpe M.; Füssel U. Analyses about the influence of the natural oxide layer of aluminium on the brazeability in a shielding gas furnace. Weld. World 2013, 57, 449–455. 10.1007/s40194-013-0031-9.

[ref10] Holländer U.; Wulff D.; Langohr A.; Möhwald K.; Maier H. J. Brazing in SiH4-Doped Inert Gases: A New Approach to an Environment Friendly Production Process. Int. J. of Precis. Eng. and Manuf-Green Tech. 2020, 7, 1059 10.1007/s40684-019-00109-1.

[ref11] Volgmann K.; Voigts F.; Maus-Friedrichs W. The interaction of H2O molecules with iron films studied with MIES, UPS and XPS. Surf. Sci. 2012, 606, 858–864. 10.1016/j.susc.2012.02.002.

[ref12] Krueger W. H.; Pollack S. R. Room temperature adsorption of water by aluminum thin films. Surf. Sci. 1972, 30, 280–298. 10.1016/0039-6028(72)90003-9.

[ref13] Lützenkirchen-Hecht D.; Wulff D.; Wagner R.; Frahm R.; Holländer U.; Maier H. J. Thermal anti-oxidation treatment of CrNi-steels as studied by EXAFS in reflection mode: the influence of monosilane additions in the gas atmosphere of a continuous annealing furnace. J. Mater. Sci. 2014, 49, 5454–5461. 10.1007/s10853-014-8257-5.

[ref14] Gustus R.; Szafarska M.; Maus-Friedrichs W. Oxygen-free transport of samples in silane-doped inert gas atmospheres for surface analysis. J. Vac. Sci. Technol., B 2021, 39, 054204 10.1116/6.0001180.

[ref15] Bulanov A. D.; Sennikov P. G.; Sozin A. Y.; Lashkov A. Y.; Troshin O. Y. Formation of impurity Si2OH6 in silane synthesized from silicon tetrafluoride. Russ. J. Inorg. Chem. 2011, 56, 510–512. 10.1134/S0036023611040061.

[ref16] Sennikov P. G.; Ignatov S. K.; Sadov A. E.; Razuvaev A. G.; Schrems O. Quantum-chemical calculation of the thermodynamics of multistep hydrolysis of MX4 molecules (M = C, Si, Ge; X = H, F, Cl) in the gas phase. Russ. J. Inorg. Chem. 2009, 54, 252–259. 10.1134/S0036023609020144.

[ref17] Mitsui Y.; Irie T.; Iijima S.; Mizokami K.; Hasumi K.; Kuriyama K. Quantitative Analysis of Trace Water in Monosilane Gas Using Atmospheric-Pressure Ionization Mass Spectrometer with Bicompartment Ion Source. Jpn. J. Appl. Phys. 1995, 34, 6308–6313. 10.1143/JJAP.34.6308.

[ref18] Hu S.-W.; Wang Y.; Wang X.-Y.; Chu T.-W.; Liu X.-Q. Gas-Phase Reactions between Silane and Water: A Theoretical Study. J. Phys. Chem. A 2004, 108, 1448–1459. 10.1021/jp036836g.

[ref19] Perrin J.; Schmitt J. P. M.; de Rosny G. D.; Drevillon B.; Huc J.; Lloret A. Dissociation cross sections of silane and disilane by electron impact. Chem. Phys. 1982, 73, 383–394. 10.1016/0301-0104(82)85177-X.

[ref20] Perkins G. G. A.; Austin E. R.; Lampe F. W. The 147-nm photolysis of monosilane. J. Am. Chem. Soc. 1979, 101, 1109–1115. 10.1021/ja00499a010.

[ref21] Bach F. W.; Möhwald K.; Holländer U. Physico-Chemical Aspects of Surface Activation during Fluxless Brazing in Shielding-Gas Furnaces. KEM 2010, 438, 73–80. 10.4028/www.scientific.net/KEM.438.73.

[ref22] Kondo S.; Tokuhashi K.; Takahashi A.; Kaise M. A Numerical Study of Low Temperature Silane Combustion. Combust. Sci. Technol. 2000, 159, 391–406. 10.1080/00102200008935792.

[ref23] Tao W.; Jung H.; Ryu T.; Hwang S.-R.; Han B. Dramatic catalytic activation of kinetically inert disilane hydrolysis in metallic iron particulate via barrierless chemical dissociation: First-principles study. Appl. Surf. Sci. 2021, 560, 149988 10.1016/j.apsusc.2021.149988.

[ref24] Paluszkiewicz C.; Jonas S.; Ptak W. S.; Walasek E. FTIR studies of the gaseous phase during CVD process. J. Mol. Struct. 1993, 294, 263–265. 10.1016/0022-2860(93)80365-3.

[ref25] George M. A. R.; Truong N. X.; Savoca M.; Dopfer O. IR Spectrum and Structure of Protonated Monosilanol: Dative Bonding between Water and the Silylium Ion. Angew. Chem., Int. Ed. Engl. 2018, 57, 2919–2923. 10.1002/anie.201712999. 29341408

[ref26] Ding L.; Marshall P. Experimental and theoretical studies of the reaction of atomic oxygen with silane. J. Chem. Phys. 1993, 98, 8545–8550. 10.1063/1.464513.

[ref27] Murakami Y.; Koshi M.; Matsui H.; Kamiya K.; Umeyama H. Kinetics of the SiH 3 + O 2 Reaction: A New Transition State for SiO Production. J. Phys. Chem. 1996, 100, 17501–17506. 10.1021/jp961249z.

[ref28] Tsai H.-Y.; Wang S.-W.; Wu S.-Y.; Chen J.-R.; Ngai E. Y.; Pai-Ping Huang K. Experimental studies on the ignition behavior of pure silane released into air. J. Loss Prev. Process Ind. 2010, 23, 170–177. 10.1016/j.jlp.2009.07.009.

[ref29] Tokuhashi K.; Horiguchi S.; Urano Y.; Iwasaka M.; Ohtani H.; Kondo S. Premixed silane-oxygen-nitrogen flames. Combust. Flame 1990, 82, 40–50. 10.1016/0010-2180(90)90076-4.

[ref30] Koda S. Kinetic aspects of oxidation and combustion of silane and related compounds. Prog. Energy Combust. Sci. 1992, 18, 513–528. 10.1016/0360-1285(92)90037-2.

[ref31] Chen J.-R.; Tsai H.-Y.; Chen S.-K.; Pan H.-R.; Hu S.-C.; Shen C.-C.; Kuan C.-M.; Lee Y.-C.; Wu C.-C. Analysis of a silane explosion in a photovoltaic fabrication plant. Process Saf. Prog. 2006, 25, 237–244. 10.1002/prs.10136.

[ref32] Saito T.; Oshima K.; Shimogaki Y.; Egashira Y.; Sugawara K.; Takahiro K.; Nagata S.; Yamaguchi S.; Komiyama H. Low Temperature Chemical Vapor Deposition of Silicon-rich Tungsten Silicide Films from Tungsten Hexafluoride–Disilane Pre-activated Mixtures. Int. J. Chem. React. Eng. 2012, 10 1 30. 10.1515/1542-6580.2666.

[ref33] Smith J. W.; Maeda Y.; Iyer R. S. Disilane-Based Low Thermal Budget Silicon Dioxide Chemical Vapor Deposition Process in a Single-Wafer Chamber. Electrochem. Solid-State Lett. 2006, 9, G141 10.1149/1.2176078.

[ref34] Arkles B. Silicon Compounds, Silanes. Kirk-Othmer Encyclopedia of Chemical Technology, 1st ed.; John Wiley & Sons, Wiley, 2000.

[ref35] Wyller G. M.; Preston T. J.; Mongstad T. T.; Lindholm D.; Klette H.; Nordseth Ø.; Filtvedt W. O.; Marstein E. S. Influence of temperature and residence time on thermal decomposition of monosilane. Energy Procedia 2017, 124, 814–822. 10.1016/j.egypro.2017.09.352.

[ref36] Ponton S.; Dhainaut F.; Vergnes H.; Samelor D.; Sadowski D.; Rouessac V.; Lecoq H.; Sauvage T.; Caussat B.; Vahlas C. Investigation of the densification mechanisms and corrosion resistance of amorphous silica films. J. Non-Cryst. Solids 2019, 515, 34–41. 10.1016/j.jnoncrysol.2019.04.005.

[ref37] Jidenko N.; Bourgeois E.; Borra J.-P. Temperature profiles in filamentary dielectric barrier discharges at atmospheric pressure. J. Phys. D: Appl. Phys. 2010, 43, 295203 10.1088/0022-3727/43/29/295203.

[ref38] Opila E. J.; Fox D. S.; Jacobson N. S. Mass Spectrometric Identification of Si-O-H(g) Species from the Reaction of Silica with Water Vapor at Atmospheric Pressure. J. Am. Ceram. Soc. 1997, 80, 1009–1012. 10.1111/j.1151-2916.1997.tb02935.x.

[ref39] Goertz V.; Weis F.; Keln E.; Nirschl H.; Seipenbusch M. The Effect of Water Vapor on the Particle Structure and Size of Silica Nanoparticles During Sintering. Aerosol Sci. Technol. 2011, 45, 1287–1293. 10.1080/02786826.2011.590555.

