# Supplementary material for: Gas Phase Reaction of Silane with Water at Different Temperatures and Supported by Plasma
Source: ACS Omega. 2023 Feb 24;8(9):8388–96. doi: 10.1021/acsomega.2c07209 (PMC9996801; doi:10.1021/acsomega.2c07209)
Supplement: Supplementary file 1 — ao2c07209_si_001.pdf [file ao2c07209_si_001.pdf]

Supporting Information

Gas phase reaction of Silane with Water at different temperatures and supported by plasma

Maik Szafarska[1, †, *], Vinzent Olszok[2, †], Ulrich Holländer[3], René Gustus[1], Alfred P. Weber[2] and Wolfgang Maus-Friedrichs[1]

† Both authors contributed equally to this work

[1] Clausthal Centre of Materials Technology, Clausthal University of Technology, Leibnizstrasse 9, 38678 Clausthal-Zellerfeld, Germany

[2] Institute of Particle Technology, Clausthal University of Technology, Leibnizstrasse 19, 38678 Clausthal-Zellerfeld, Germany

[3] Institut für Werkstoffkunde (Materials Science), Leibniz Universität Hannover, An der Universität 2, 30823 Garbsen, Germany

* Corresponding Author Email: maik.szafarska@tu-clausthal.de

Corresponding EDS spectra for the quantitative results shown in TABLE I regarding the TEM micrographs of SiO_x particles.

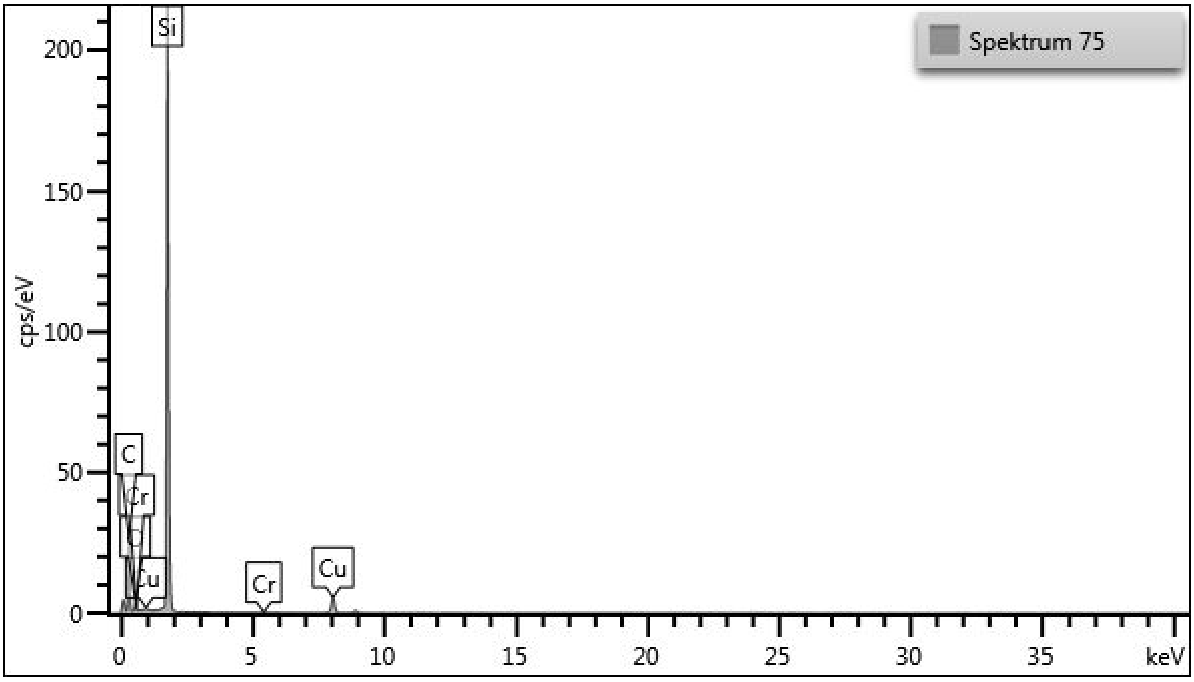

Figure S1: Corresponding EDS spectrum to TEM micrograph a).

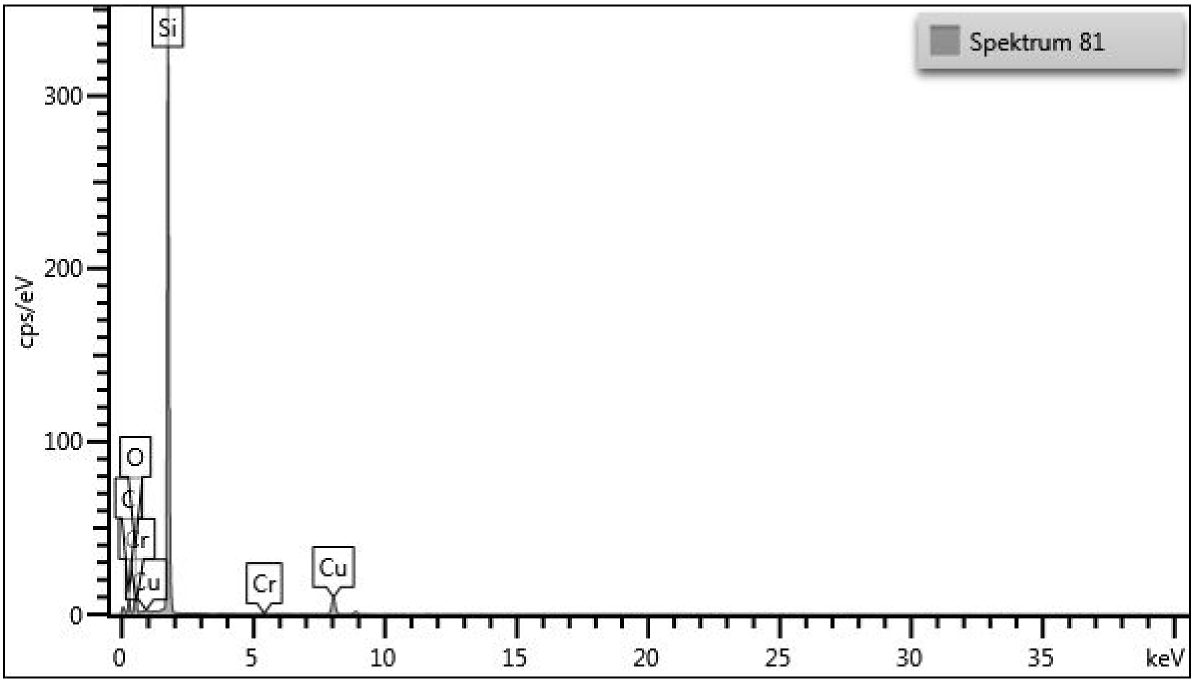

Figure S2: Corresponding EDS spectrum to TEM micrograph b).

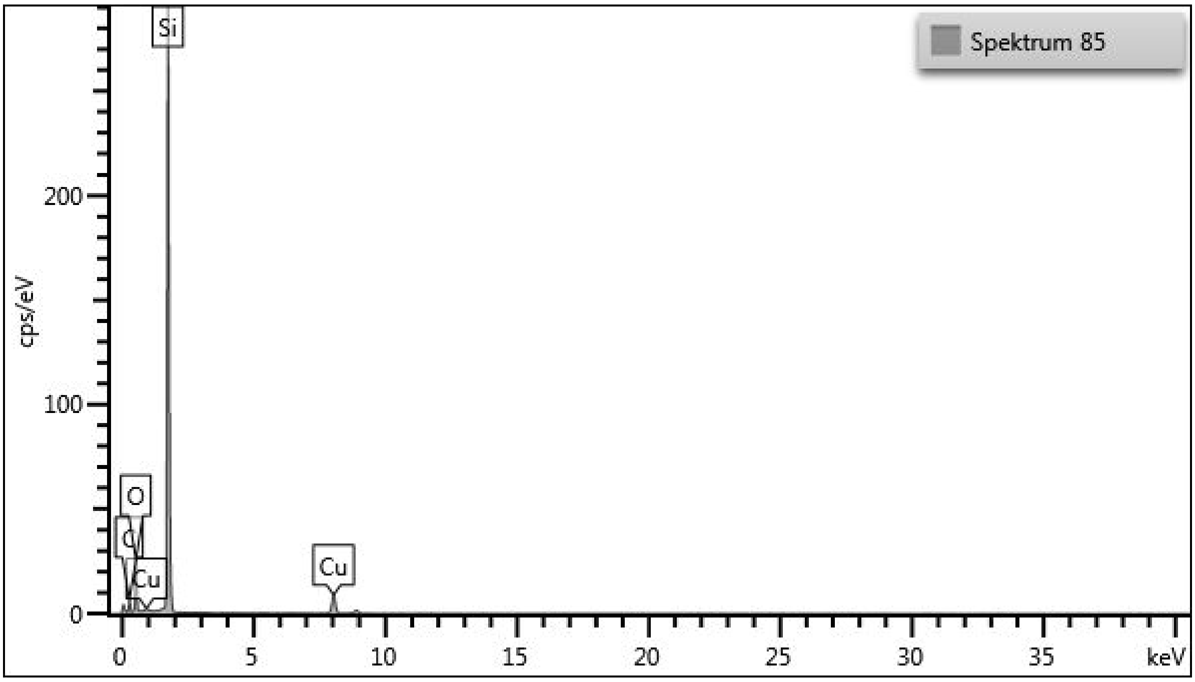

Figure S3: Corresponding EDS spectrum to TEM micrograph c).

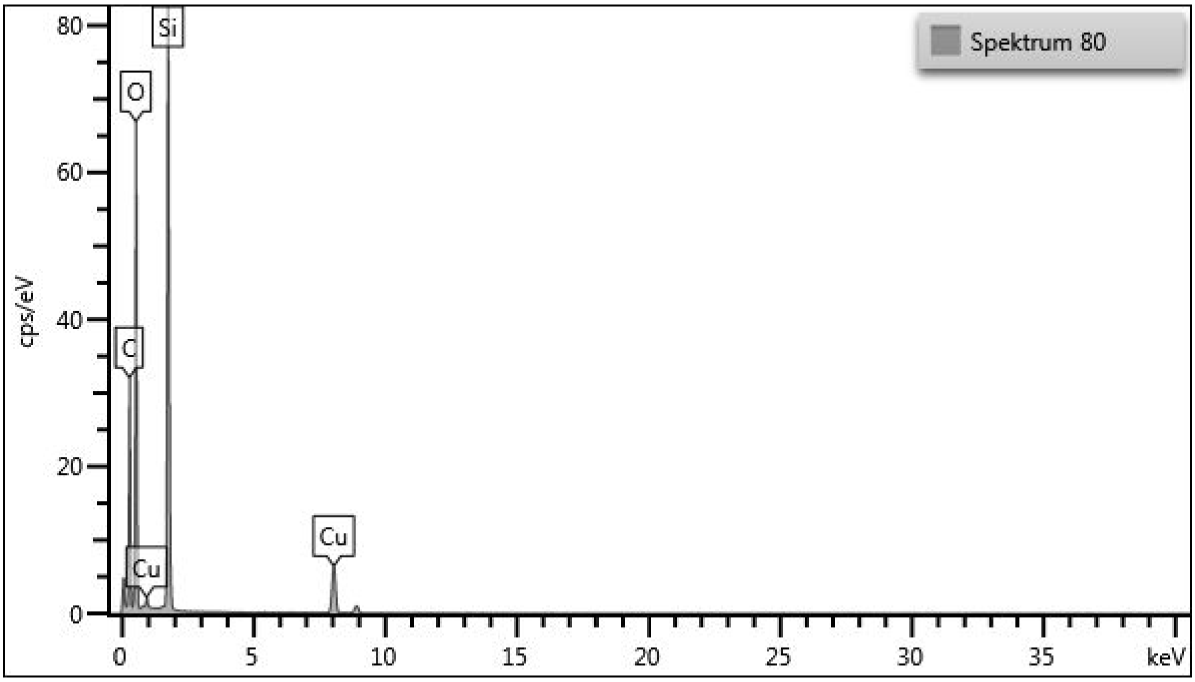

Figure S4: Corresponding EDS spectrum to TEM micrograph d).
